# Association of Extravascular Leakage on Computed Tomography Angiography with Fibrinogen Levels at Admission in Patients with Traumatic Brain Injury

**DOI:** 10.1089/neur.2022.0054

**Published:** 2022-12-26

**Authors:** Hiroshi Ito, Youhei Nakamura, Yuki Togami, Shinya Onishi, Shunichiro Nakao, Jiro Iba, Hiroshi Ogura, Jun Oda

**Affiliations:** Department of Traumatology and Acute Critical Medicine, Osaka University Graduate School of Medicine, Osaka, Japan.

**Keywords:** contrast-enhanced computed tomography, extravascular leakage, fibrinogen, traumatic brain injury

## Abstract

Extravascular leakage on computed tomography (CT) angiography in patients with traumatic brain injury (TBI) is associated with hematoma expansion, functional prognosis, subsequent surgery, and death. Fresh frozen plasma (FFP) administration is often necessary to treat coagulation disorders associated with TBI. This study aimed to determine the relationship between the presence of extravascular leakage on contrast-enhanced head CT, fibrinogen level at admission, and FFP administration in patients with TBI. The medical records of patients with TBI ≥18 years of age referred to our hospital between January 2010 and December 2020 were examined retrospectively. Patients who underwent contrast-enhanced CT immediately after admission were selected, and the presence or absence of extravascular leakage, fibrinogen level at admission, and percentage of patients who required FFP administration within 24 h of admission were examined; 172 patients were included. Multi-variable linear regression analysis was performed to determine the effects of contrast extravasation on fibrinogen levels at admission and was adjusted for age, sex, systolic blood pressure, time from injury to admission, Marshall CT score, Glasgow Coma Scale score at admission, Injury Severity Score, and need for emergency surgery; the regression coefficient was −19.8. The effect of extravasation on FFP administration within 24 h of admission was analyzed using logistic regression while adjusting for age, systolic blood pressure, Marshall CT score, need for emergency surgery, and fibrinogen level at admission. The odds ratio of contrast extravasation was 7.08 after adjustment. Extravascular leakage is associated with fibrinogen levels at admission and FFP administration within 24 h of admission.

## Introduction

Coagulation disorders can cause secondary brain damage after traumatic brain injury (TBI), and it is crucial to avoid such sequelae in severe TBI. Coagulation disorders are observed in ∼20% of patients with TBI.^1^ Coagulation and fibrinolytic system disorders are associated with a 10-fold increased risk of death and poor prognosis in TBI.^2,3^ Degree of coagulation disorders can be recognized using blood tests, and therapeutic interventions, such as blood component replacement therapy, are well established. Abnormal levels of fibrinogen could be an indicator of impaired coagulation and fibrinolysis. In the early stages of trauma, fibrinogen consumption increases while its synthesis remains constant, resulting in extremely low levels of available fibrinogen.^4,5^ A previous study showed that low fibrinogen levels were common in patients with TBI: 38.6% and 15.7% of patients had fibrinogen levels <200 and <150 mg/dL, respectively, which indicate severe hypofibrinogenemia. Low fibrinogen levels at admission were also associated with poor prognosis in patients with TBI.^6^

European trauma guidelines recommend fresh frozen plasma (FFP) transfusion for trauma patients if the prothrombin time/international normalized ratio (PT-INR) and/or activated partial thromboplastin time (aPTT) is at least 1.5 times the normal value,^7^ especially in patients with TBI, for whom fibrinogen levels should be maintained above 150 mg/dL.^8^ Currently, three fibrinogen sources are available to clinicians: fibrinogen concentrate, FFP, and cryoprecipitate.^9^ The Collaborative European Neurotrauma Effectiveness Research in Traumatic Brain Injury study reported that 73% of centers use FFP transfusion for treating TBI-related coagulopathy, whereas 52% use platelet transfusion.^10^ Considering the widespread use of FFP transfusion, indications for this therapy should be clearly determined during the management of TBI with coagulopathy. It is necessary to determine the administration of FFP in TBI with coagulopathy, and recognition of post-injury fibrinogen levels may be very important.

Computed tomography (CT) of the head is often performed on patients with TBI in the emergency department, and its findings can directly support surgical decision making. In addition, computed tomography angiography (CTA) is widely used as an initial screening test for the detection of traumatic vascular injuries, and the French Society of Anesthesia, Critical Care, and Perioperative Medicine guidelines recommend the use of CTA to examine intracranial vessels in patients with risk factors.^11^ Extravascular leakage observed on CTA scans of patients with TBI is associated with hematoma expansion, functional prognosis, need for surgery, and death.^12–15^ Extravascular leakage on CTA can help identify patients with hematoma expansion and high mortality risk, thus influencing their management in the intensive care unit and subsequent treatment strategies.^16^ In addition to the hematoma itself, hematoma expansion in TBI might be associated with fibrinogen depletion. However, no study has evaluated the relationship between extravascular leakage in TBI, fibrinogen level at admission, and FFP administration within 24 h of admission.

The purpose of this study was to evaluate the association between the presence of extravascular leakage on contrast-enhanced CT images of patients with TBI, fibrinogen level at admission, and FFP administration within 24 h of admission.

## Methods

### Study design

This was a single-center, retrospective, observational study.

### Setting

This study was conducted from January 2010 to December 2020 and was approved by the ethics committee of our hospital (approval no.: 21240). The study site was the Department of Traumatology and Acute Critical Medicine, Graduate School of Medicine, Osaka University. Approximately 200–300 trauma cases are transported to the center annually. In Japan, emergency medical service personnel can administer only fluid to trauma patients who are in shock. They cannot intubate and administer paralytics or sedation.

The emergency department of the hospital is equipped with a CT scanner and a full-time radiological technologist. Per the center's established protocol, a contrast-enhanced CT scan of the head is performed in the following cases: 1) intracranial hemorrhage, facial bone fracture, or skull fracture revealed on plain CT scan; 2) Glasgow Coma Scale (GCS) score ≤8 points on arrival attributable to TBI; 3) neurological abnormalities unexplained on a plain CT scan; and 4) cervical spine or cervical cord injury identified on plain CT scan.

All examinations were conducted using a 64-section CT scanner (SOMATOM Definition Flash; Siemens, Erlangen, Germany). A plain CT was performed after patients were stabilized by treating physicians. Contrast-enhanced CT scans were performed on the ascending aorta or skull base in the parietal region. Nonionic contrast material (95 mL, iopamidol) was administered at 4 mL/s by using a power injector. The radiological technologist visually confirmed the tracking section targeting the internal carotid artery and started imaging 2 sec after the CT number had reached ∼100 Hounsfield units (HU). Six seconds after the first phase completion, the second phase was initiated. Plain CT images were taken with single-energy CT (SECT), and contrast CT images were obtained with either SECT or dual-energy CT (DECT). CT imaging was performed according to the scan parameters described in the Supplementary Materials.

Plain CT images were reconstructed with a 0.6-mm slice thickness. The original DECT data set was automatically reconstructed, using an adaptive iterative reconstruction algorithm (ADMIRE; Siemens) with a strength value of 2 and a slice thickness of 0.6 mm, and then sent to the picture archiving and communication system.

The need for FFP administration in TBI treatment at our center is determined by the treating physicians and is based on patient vital signs and a comprehensive assessment of intraoperative findings. FFP was administered to both surgical and non-surgical patients alike, with monitoring to ensure that fibrinogen levels did not fall below 150 mg/dL.

Regarding informed consent, we disclosed information concerning this study on the basis of an opt-out approach.

### Participants

Patients ≥18 years of age with isolated TBI (Abbreviated Injury Scale [AIS] score <3 other than the head) who were sent directly to our hospital without cardiopulmonary arrest were included. Inclusion criteria were acute intracranial hemorrhage that was not a chronic subdural hematoma, as indicated by a plain CT scan at admission. Exclusion criteria included patients who did not undergo a contrast CT immediately after arrival, underwent a contrast CT of the head by a method other than that described in the *Setting* section, and refused surgery or intensive care. Patients whose fibrinogen levels were not measured at admission were excluded.

### Variable

The following information was obtained from the medical records: age, sex, history of antiplatelet or -coagulant therapy, mechanism of injury, GCS at admission, anisocoria at admission, blood pressure at admission, time of injury, time of admission, start time of contrast CT head scan, injury sites, AIS, Injury Severity Score (ISS), need for emergency surgery, Marshall CT score, hematoma morphology considering the main pathology, blood transfusion volume within 24 h of admission, and Glasgow Outcome Scale (GOS) score at discharge were collected using a standardized data collection sheet. In this study, shock was defined as systolic blood pressure under 90 mm Hg.^17,18^ Marshall CT score was calculated from CT images, and the appropriate GOS was selected from nursing and rehabilitation records in the medical record.

Blood laboratory values immediately after admission included platelet count, PT-INR, aPTT, fibrinogen level, fibrinogen degradation product (FDP) level, and D-dimer level. Fibrinogen values were also recorded as the lowest value among those measured within 24 h of hospital admission. PT-INR, aPTT, and fibrinogen values were measured using the coagulation method by the Thromborel S^®︎^, Thrombocheck APTT-SLA^®︎^, and Thrombocheck Fib(L)^®︎^ (Sysmex, Kobe, Japan), respectively. FDP and D-dimer levels were measured using turbidimetric immunoassay by the NANOPIA P-FDP^®︎^ (Sekisui Medical, Tokyo, Japan) and LPIA-ACE D-dimer II (LSI Medience, Tokyo, Japan), respectively. An immunoassay was performed using the LPIA-ACE D-dimer II (LSI Medience).

The primary outcome was fibrinogen level at admission, and the secondary outcome was the percentage of patients who received FFP within 24 h.

The need for emergency surgery in this study was decided by the physician in charge after a comprehensive assessment of the plain CT scan of the head at the patient's arrival and clinical symptoms. In this study, emergency surgery was defined as neurosurgical operations deemed necessary to be performed immediately after a plain CT scan of the head after arrival at the hospital. Surgery necessary owing to worsening symptoms under observation was not included in emergency surgery. TBI treatment at our center was based on the guidelines for TBI management by the Japan Society of Neurotraumatology.^19–21^ Supplementary Tables S1 and S2 show the Marshall CT score^22^ and Glasgow Outcome Scale (GOS) definition.^23^

One interventional radiologist (S.O.), one neurosurgeon (Y.N.), and two emergency physicians (H.I., Y.T.) examined contrast CT scans. All contrast-enhanced CT scans were evaluated together with plain CT scans because the presence of bone fragments and other objects could be misinterpreted as an extravascular invasion. Images were assessed blinded to the medical records. Images were read in 0.6-mm slices. The following definitions from previous studies were used to identify extravascular leakage in the images^14^: 1) one or more contrast reservoirs within the hematoma; 2) discontinuity with adjacent vessels; 3) >120 HU; and 4) any size or morphology. In the current study, extravasation was defined as “contrast extravasation” when extravasation was observed in either phase 1 or 2 and as “no contrast extravasation” when extravasation was not observed in either phase. One physician (H.I.) first read all the images. Next, targeted images were assigned to three physicians (S.O., Y.N., and Y.T.) and read by them. Images that did not agree with H.I.'s reading were read by one of the other two of the three (Y.T., Y.N., and S.O.), and the reading findings that were agreed upon were adopted.

### Statistical analyses

Summary data are presented as medians (interquartile range; IQR) for continuous variables and numbers (%) for categorical variables. The Mann–Whitney U test was used for continuous variables, and the chi-square test and Fisher's exact test were used for binary variables. Inter-reader agreement (H.I. vs. Y.N., H.I. vs. S.O., and H.I. vs. Y.T.) was calculated using kappa values. Uni- and multi-variable linear regression analyses of the effect of contrast extravasation on fibrinogen levels at admission were performed to calculate the regression coefficient and standard error for each variable. The response variable was the fibrinogen level at admission, and the explanatory variable was the presence or absence of contrast extravasation. Adjustment factors were selected from items that differed in the background of the presence or absence of contrast extravasation (*p* < 0.2), items that were related to fibrinogen level in accordance with previous studies^24–29^ (age, sex systolic blood pressure, time from onset to arrival, and ISS), and items that assessed the severity of TBI (admission GCS ≤8, Marshall CT score, and AIS-head).^30^ Items with high covariance were excluded. Model diagnosis was performed as drawing a scatter plot and Quantile-Quantile (QQ) plot of the residuals and testing normality of residuals with the Shapiro-Wilk normality test. Correlation analyses were also performed to calculate the correlation coefficient and 95% confidence intervals (CIs).

To evaluate the effect of contrast extravasation on FFP administration within 24 h, the response variable was FFP administration within 24 h, and the explanatory variable was the presence of contrast extravasation. Fibrinogen levels were added to the above adjustment factors, and items that were clinically important and associated with FFP administration were selected as adjustment factors. Uni- and multi-variable logistic regression analyses were performed to calculate the odds ratios (ORs) and 95% CIs.

Background factors between the two groups of patients with and without contrast extravasation were detected in patients who underwent emergency surgery.

Statistical analyses were performed using commercially available statistical analysis software (JMP Pro 16 software; SAS Institute Inc., Cary, NC). Statistical significance was set at *p* < 0.05.

## Results

**FIG. 1.**
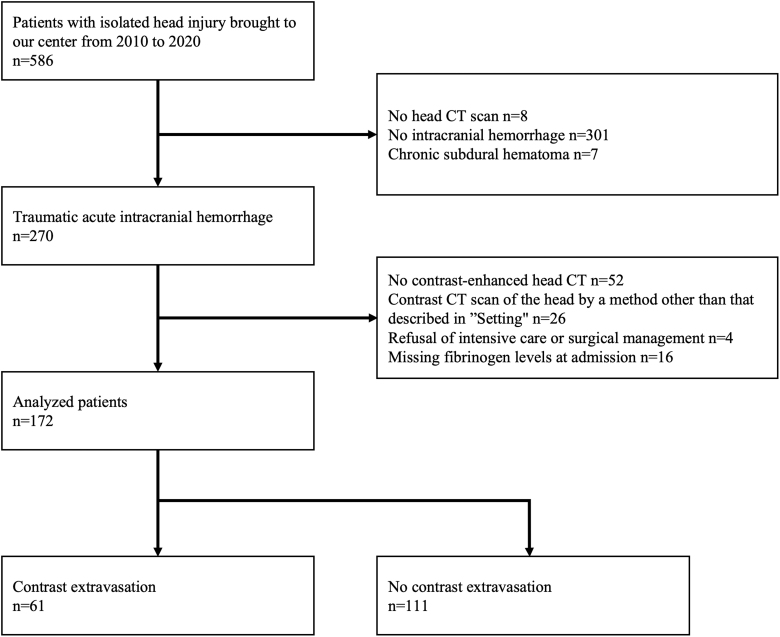
Patient flow chart. A total of 172 patients were included in the analysis. CT images of 61 and 111 patients show contrast extravasation and no contrast extravasation, respectively. CT, computed tomography.

Overall, 270 patients met the inclusion criteria. In total, 172 patients (61 had contrast extravasation and 111 patients had no contrast extravasation) were included in the analyses, and the remaining patients were excluded (Fig. 1).

Median age of the 172 included patients was 65.5 years, and 111 patients (64.5%) were men. The forms of intracranial hematoma were acute subdural hematoma (ASDH), acute epidural hematoma (AEDH), intracerebral hemorrhage (ICH), and traumatic subarachnoid hemorrhage (tSAH) in 72 (41.9%), 13 (7.56%), 28 (16.3%), and 58 (33.7%) patients, respectively. Emergency surgery was required in 68 patients (39.5%), median GOS score at discharge was 3, and 31 patients died in the hospital (18.0%; Table 1).

**Table 1.** Clinical and Radiological Characteristics Between Patients with Contrast Extravasation and with No Contrast Extravasation

Characteristic	Total (*n* = 172)	Contrast extravasation (*n* = 61)	No contrast extravasation (*n* = 111)	*p* value
Age, median (IQR), years	65.5 (47–76)	69 (53.0–76.5)	63 (43–76)	0.14
Male sex, no. (%)	111 (64.5)	43 (70.5)	68 (61.3)	0.23
Patients taking anticoagulant medicine, no. (%)	14 (8.14)	4 (6.56)	10 (9.0)	0.77
Patients taking antiplatelet medicine, no. (%)	16 (9.3)	4 (6.56)	12 (10.8)	0.42
Mechanism of injury, no. (%)				0.41
Fall from standing	40 (23.3)	15 (24.6)	25 (22.5)	
Fall from height	30 (17.4)	14 (23.0)	16 (14.4)	
Motor vehicle crash	38 (22.1)	9 (14.8)	29 (26.1)	
Bicycle accident	44 (25.6)	17 (27.9)	27 (24.3)	
Pedestrian struck	18 (10.5)	6 (9.84)	12 (10.8)	
Hit by object	2 (1.16)	0 (0)	2 (1.8)	
Admission blood pressure, median (IQR), mm Hg				
Systolic	153 (133–181)	159 (138.0–191.5)	149.5 (130–174)	0.14
Diastolic	92 (80–107)	91 (80–109)	92.5 (77.8–105.5)	0.80
Shock, no. (%)	4 (2.32)	0 (0)	4 (3.6)	0.30
Admission GCS, median (IQR)	11 (6–14)	11 (6–14)	12 (6–14)	0.37
Admission anisocoria, no. (%)	40 (25.6)	15 (25.0)	25 (26.0)	0.88
Time from onset to arrival, median (IQR), min	35 (28–44)	35 (29–44)	35 (27–44)	0.75
Time from onset to CTA, median (IQR), min	64 (56–75)	64 (56–74)	64 (56–80)	0.66
Marshall Computed Tomography Score, median (IQR)	2 (2–5)	4 (2.0–5.5)	2 (2–5)	0.0014
Type of traumatic intracranial hemorrhage, no. (%)				<0.001
ASDH	72 (41.9)	26 (42.6)	46 (41.4)	
AEDH	13 (7.56)	10 (16.4)	3 (2.7)	
ICH	28 (16.3)	18 (29.5)	10 (9.0)	
tSAH	58 (33.7)	7 (11.5)	51 (45.9)	
IVH	1 (0.58)	0 (0)	1 (0.9)	
AIS-head, median (IQR)	5 (4–5)	5 (5–5)	4 (3–5)	<0.0001
ISS, median (IQR)	25 (17–26)	25 (25–29)	21 (16–26)	<0.0001
Emergent surgery, no. (%)	68 (39.5)	33 (54.1)	35 (31.5)	0.0038
GOS at discharge, median (IQR)	3 (2–4)	2 (1.0–3.5)	3 (3–4)	<0.0001
In-hospital mortality, no. (%)	31 (18.0)	22 (36.1)	9 (8.11)	<0.0001

IQR, interquartile range; GCS, Glasgow Coma Scale; CTA, computed tomography angiography; ASDH, acute subdural hematoma; AEDH, acute epidural hematoma; ICH, intracerebral hemorrhage; tSAH, traumatic subarachnoid hemorrhage; IVH, intraventricular hemorrhage; AIS, Abbreviated Injury Scale; ISS, Injury Severity Score; GOS, Glasgow Outcome Scale.

Clinical characteristics were compared between patients with and without contrast extravasation. Kappa values for inter-reader agreements were 0.64 (HI vs. YN), 0.65 (HI vs. SO), and 0.85 (HI vs. YT). Among patients with AEDH and ICH, contrast extravasation was observed more frequently than no contrast extravasation. Among patients with tSAH, no contrast extravasation was observed more frequently than contrast extravasation. Cases with contrast extravasation required more emergency surgeries (54.1% vs. 31.5%; *p* = 0.0038), had worse GOS scores at discharge (2 vs. 3; *p* < 0.0001), and had higher in-hospital mortality rates (36.1% vs. 8.11%; *p* < 0.0001) than cases without contrast extravasation. Patients with contrast extravasation had significantly lower fibrinogen levels at admission (203 vs. 251 mg/dL; *p* = 0.0007), which was the primary outcome. The proportion of patients who required FFP within 24 h of presentation, which is the secondary outcome, was high (59.0% vs. 18.9%; *p* < 0.0001; Table 2). Minimum fibrinogen level within 24 h of hospital admission (148 vs. 234 mg/dL; *p* < 0.0001) and platelet level at admission (197 vs. 220 × 10^3^/μL; *p* = 0.01) were lower in patients with contrast extravasation, and the aPTT at admission (29 vs. 27 sec; *p* = 0.03), FDP (92.2 vs. 25.3 μg/mL; *p* < 0.0001), and D-dimer (24.0 vs. 8.69 μg/mL; *p* < 0.0001) at admission were higher in patients with contrast extravasation (Table 2).

**Table 2.** Differences of Blood Test and Transfusion Between Contrast Extravasation and No Contrast Extravasation

Outcome	Contrast extravasation (*n* = 61)	No contrast extravasation (*n* = 111)	*p* value
Primary outcome			
Admission fibrinogen, median (IQR), mg/dL	203 (167.5–260.5)	251 (205–303)	0.0007
Secondary outcome			
No. of patients within 24 h of fresh frozen plasma transfusions, no. (%)	36 (59.0)	21 (18.9)	<0.0001
Minimum fibrinogen level within 24 h of admission, median (IQR), mg/dL	148 (102.5–209.5)	234 (184–273)	<0.0001
Admission platelet count, median (IQR), × 10^3^/μL	197 (152.5–235)	220 (176–257)	0.01
Admission PT-INR, median (IQR)	1.08 (1.01–1.18)	1.06 (1.01–1.13)	0.22
Admission aPTT, median (IQR), sec	29 (25–35)	27 (25–30)	0.03
Admission FDP, median (IQR), μg/mL	92.2 (47.8–256.0)	25.3 (9.7–52.5)	<0.0001
Admission D-dimer, median (IQR), μg/mL	24.0 (15.3–64.8)	8.69 (3.19–18.5)	<0.0001
No. of patients within 24 h of transfusions, no. (%)			
Red blood cell	32 (52.5)	21 (18.9)	<0.0001
Platelet concentrate	14 (23.0)	3 (2.7)	<0.0001
No. of units within 24 h of transfusions, median (IQR), units			
Red blood cell	2 (0–10)	0 (0–0)	<0.0001
Fresh frozen plasma	6 (0–16)	0 (0–0)	<0.0001
Platelet Concentrate	0 (0–0)	0 (0–0)	<0.0001

IQR, interquartile range; PT-INR, prothrombin time/international normalized ratio; aPTT, activated partial thromboplastin time; FDP, fibrinogen degradation products.

Items with differences (*p* < 0.2) in the background of the presence or absence of extravasation included age, systolic blood pressure, Marshall CT score, type of traumatic intracranial hemorrhage, AIS-head, ISS, and emergent surgery. Because type of traumatic intracranial hemorrhage and AIS-head had high covariance with Marshall CT score as an assessment of brain damage morphology, they were excluded. Adding factors that were related to fibrinogen level and severity of TBI, age, sex, systolic blood pressure, admission GCS (≦8), time from onset to arrival, Marshall CT score, ISS, and emergent surgery were selected as adjustment factors.

Univariable linear regression analysis showed a regression coefficient of −16.6 (standard error, 6.38; *p* = 0.0099) for contrast extravasation. Multi-variable linear regression analysis resulted in an estimate of −19.8 (standard error, 7.02; *p* = 0.0055). Correlation analysis resulted in a correlation coefficient of −0.2 (95% CI, 0.048–0.340; *p* = 0.0099; Table 3). Presence of contrast extravasation was associated with fibrinogen levels, which were 19.8 mg/dL lower than those without contrast extravasation. Model diagnostics for this regression model were performed. Scatter plots of residuals showed normal distribution, and QQ plots were on a straight line (Supplementary Figs.S1 and S2). This data set was well modeled by a normal distribution (*p* < 0.0001).

**Table 3.** Results of Uni- and Multi-Variable Linear Regression Analyses and Correlation Analyses Assessing the Impact of Clinical Parameters, Including Contrast Extravasation, on Admission Fibrinogen Levels

Variables	Univariable analysis			Multi-variable analysis			Correlation analysis			
	Estimate	Standard error	*p* value	Estimate	Standard error	*p* value	Correlation coefficient	Lower 95% CI	Upper 95% CI	*p* value
Contrast extravasation	–16.6	6.38	0.0099	–19.8	7.02	0.0055	–0.2	0.048	0.34	0.0099
Age	1.06	0.3	0.0006	1.06	0.32	0.0012	0.26	0.12	0.39	0.0006
Male/female sex	5.28	6.49	0.42	4.57	6.35	0.47	–0.062	–0.21	0.088	0.42
Systolic blood pressure	0.58	0.18	0.0013	0.54	0.18	0.0027	0.25	–0.094	0.39	0.28
Admission GCS (≦8)	–5.28	6.49	0.42	–7.97	8.71	0.36	0.062	–0.088	0.21	0.42
Time from onset to arrival, min	0.23	0.09	0.013	0.15	0.09	0.11	0.19	0.04	0.33	0.013
Marshall CT score	–9.35	3.73	0.013	–9.58	5.38	0.077	–0.19	–0.33	–0.04	0.013
ISS	–0.96	0.92	0.3	0.54	1.05	0.61	–0.08	–0.23	0.071	0.3
Emergent surgery	–9.51	6.32	0.13	0.76	10.1	0.11	0.11	–0.036	0.26	0.13

GCS, Glasgow Coma Scale; CT, computed tomography; ISS, Injury Severity Score; CI, confidence interval.

Logistic regression analysis of the effect of contrast extravasation on FFP administration within 24 h of admission showed an OR of 6.17 (95% CI, 3.07–12.40; *p* < 0.0001) for contrast extravasation before adjustment. Contrast extravasation, age, systolic blood pressure, Marshall CT score, emergent surgery, and fibrinogen levels on admission were selected as adjustment factors. Multi-variable logistic regression analysis showed an OR of 7.08 (95% CI, 2.38–21.10; *p* = 0.0004) after adjustment (Table 4).

**Table 4.** Results of Uni- and Multi-Variable Logistic Regression Analyses Assessing the Impact of Clinical Parameters, Including Contrast Extravasation, on Fresh Frozen Plasma Transfusions Within 24 h of Admission

Variables	Univariable analysis				Multi-variable analysis			
	OR	Lower 95% CI	Upper 95% CI	*p* value	OR	Lower 95% CI	Upper 95% CI	*P* value
Contrast extravasation	6.17	3.07	12.4	<0.0001	7.08	2.38	21.1	0.0004
Age	1.02	1.00	1.04	0.022	1.02	0.99	1.05	0.12
Systolic blood pressure	1.01	1.00	1.02	0.12	1.00	0.99	1.01	0.96
Marshall CT score	2.61	2.02	3.36	<0.0001	2.11	1.47	3.03	<0.0001
Emergent surgery	12.8	5.97	27.6	<0.0001	3.5	1.11	11.1	0.033
Fibrinogen levels on admission	0.99	0.99	1.00	0.0008	0.99	0.99	1.00	0.057

CT, computed tomography; OR, odds ratio; CI, confidence interval.

A comparison between patients with and without contrast extravasation who underwent emergency surgery showed significant differences in fibrinogen level at admission, minimum fibrinogen level within 24 h of admission, percentage of patients who required a blood transfusion, and amount of blood transfused (Supplementary Table S3).

## Discussion

Contrast extravasation in TBI was associated with lower fibrinogen levels at admission, and such cases may have a greater need for FFP within 24 h than cases with no contrast extravasation.

### Fibrinogen

This study showed that fibrinogen levels at admission were lower with contrast extravasation than without contrast extravasation (Table 3). Moreover, contrast extravasation was associated with low fibrinogen levels on admission. Contrast extravasation was associated with lower minimal fibrinogen levels during treatment. FDP and D-dimer levels at presentation were higher in contrast extravasation cases than in no contrast extravasation cases, although this comparison was between the two groups (Table 2).

A report on TBI-induced coagulopathy indicated that fibrinolytic D-dimer and FDPs are first detected within minutes after injury, and that prolonged prothrombin and partial thromboplastin times peak at ∼3–6 h after TBI.^31^ Moreover, D-dimer, which is a fibrinolytic factor, is increased in patients with TBI, with increased fibrinogen consumption during the acute phase of TBI.^32^ Our results are consistent with previous literature, and suggest that the patients with contrast extravasation may have had more severe TBI-induced coagulopathy.

Results also suggest that evaluating contrast extravasation in contrast-enhanced CT may be useful for the early recognition of coagulopathy. It takes time to obtain fibrinogen results from blood tests, having been reported as taking 88 min (median) to obtain coagulation test results.^33^ Waiting for fibrinogen-level results before deciding on treatment for coagulation disorders may cause delays in therapeutic intervention. At our center, time from arrival to head CTA was ∼30 min according to results in Table 1, thus allowing for early evaluation after the patient arrives at the hospital. It is important to recognize contrast extravasation on contrast-enhanced CT scans as early as possible after injury and strategize the appropriate treatment accordingly, given that contrast extravasation may emphasize to be more careful about fibrinogen levels.

### Fresh frozen plasma administration

This study suggests that contrast extravasation observed on diagnostic imaging may indicate the need for FFP administration within 24 h of admission. Table 2 shows that patients with contrast extravasation tended to receive more FFP than those without contrast extravasation. Coagulopathy is often observed in severe TBI,^1^ and FFP administration is often required to provide coagulation factor replacement because of excessive bleeding, especially in patients who require surgery. In fact, in the present study, the comparison between the two groups of patients who underwent emergency surgery showed that those with contrast extravasation required more blood transfusions (Table S3). Although early FFP administration has been associated with improved hospital survival in isolated TBI with multiple brain hemorrhages,^34^ there have been reports of an association between early FFP administration and the risk of developing multiple organ failure and acute respiratory distress syndrome (ARDS) in trauma patients.^35^ Although the causal relationship between FFP and ARDS has not been clarified, FFP should be administered with caution. Future studies should prospectively examine whether contrast extravasation is associated with low fibrinogen levels and the patient characteristics for whom contrast-enhanced CT of the head are beneficial.

### Future prospects

Contrast-enhanced CT is necessary for evaluating contrast extravasation in patients with TBI; nonetheless, debate exists on the types of patients with TBI who would benefit from this assessment based on characteristics, like pre-hospital information, findings at admission, and initial plain CT scan.

In this study, we included cases of isolated TBI assessed using the same imaging method. Whole-body CT is shown to be effective in cases of multiple traumas,^36^ and there have been cases in which whole-body CT was performed at our center after treating-physician–suspected multiple trauma according to pre-hospital information and physical examination findings. In some cases, the head was included in the contrast area. In the future, consideration should be given to whether any information on indicators for FFP use can be obtained even in patients with TBI with multiple trauma or when the head is included in the contrast area of whole-body CT.

### Limitations

This study has limitations. First, this was a single-center, retrospective, observational study of medical records with a convenience sample of patients who underwent CTA. In addition, FFP was administered at the discretion of the treating physician, not in a rigorous protocolized fashion, which could have influenced the results concerning FFP administration. There were some cases in which patients had intracranial hemorrhage, but contrast CT was not performed at the discretion of the physician in charge; this may have caused selection bias. Moreover, contrast CT was not performed for patients with minor hemorrhage on plain CT scans and with good consciousness.

Second, contrast-enhanced CT examinations were performed using either DECT or SECT; however, the modality was chosen according to the skill of the radiological technologist in charge. This may have affected the accuracy in identifying contrast extravasation.

Finally, besides the three adjustment factors used in the multi-variable logistic analysis, many other factors may have influenced the administration of FFP within 24 h of the patient's arrival at the hospital, including the amount of fluid administered, time of FFP administration, infusion rate of FFP, and type of surgery. The sample size for this study was small to adjust for all these factors, and extraction of all relevant confounding factors in detail from medical records was difficult.

## Conclusion

This study suggests that extravascular leakage observed in contrast-enhanced CT of the head is associated with lower fibrinogen levels at presentation and those with extravascular leakage may more frequently require FFP within 24 h.

## Supplementary Material

Supplemental data

Supplemental data

Supplemental data

Supplemental data

Supplemental data

Supplemental data

## Transparency, Rigor, and Reproducibility Summary

This study was not formally registered because it is a retrospective, observational study without any intervention. The analysis plan was not formally pre-registered. To obtain data, the medical records of 1247 patients were screened based on their TBI diagnosis, and 1193 patients had actual TBI confirmed by review of their medical records. This study included 586 patients ≥18 years of age with an isolated TBI, and 188 patients were included in the analyses. Fibrinogen levels, the primary end-point, were not measured on admission for 16 patients. All patients were evaluated for transfusion administration, course of hospitalization, and GOS score at discharge. Final clinical outcome assessments and judgments were made by team members with knowledge of the relevant characteristics of the participants. Data were collected between April 2021 and February 2022 by using a standardized data collection sheet. Analyses were performed using commercially available statistical analysis software (JMP Pro 16 software). The time required for data acquisition was 8 months. The time required for pre-processing and analysis was 4 months. All data are available from the authors. The key inclusion criteria (the presence of extravascular leakage on head contrast CT scan) is a well-established imaging finding in the field of stroke, but has not been reported in the field of TBI. Clinical outcome measures such as fibrinogen level and GOS score are well established. CIs have been reported in the abstract for the primary outcome and the main text for all outcomes. Statistical analyses and review were performed by Hiroshi Ito (first author) and Shunichiro Nakao, who is certified (MSc epidemiology). Replication by the study group is ongoing. Deidentified data from this study are not available in a public archive. Deidentified data from this study will be made available (as allowable according to the institutional review board standards) starting from August 1, 2024, by e-mailing the corresponding author. There is no analytical code associated with this study. The authors agree to provide the full content of the manuscript on request by contacting the corresponding author.

## Abbreviations Used

AEDH: acute epidural hematoma
AIS: Abbreviated Injury Scale
aPTT: activated partial thromboplastin time
ARDS: acute respiratory distress syndrome
ASDH: acute subdural hematoma
CI: confidence interval
CT: computed tomography
CTA: computed tomography angiography
DECT: dual-energy CT
FDP: fibrinogen degradation products
FFP: fresh frozen plasma
GCS: Glasgow Coma Scale
GOS: Glasgow Outcome Scale
HU: Hounsfield unit
ICH: intracerebral hemorrhage
IQR: interquartile range
ISS: Injury Severity Score
OR: odds ratio
PT-INR: prothrombin time/international normalized ratio
QQ: Quantile-Quantile
SECT: single-energy CT
tSAH: traumatic subarachnoid hemorrhage
TBI: traumatic brain injury

## References

[1] Böhm JK, Güting H, Thorn S, et al. Global characterisation of coagulopathy in isolated traumatic brain injury (iTBI): CENTER-TBI participants and investigators. Neurocrit Care 2021;35(1):184–196; doi: 10.1007/s12028-020-01151-7 33306177 PMC8285342

[2] Carrick MM, Tyroch AH, Youens CA, et al. Subsequent development of thrombocytopenia and coagulopathy in moderate and severe head injury: support for serial laboratory examination. J Trauma 2005;58(4):725–729; discussion, 729; doi: 10.1097/01.TA.0000159249.68363.78 15824648

[3] van Gent JAN, van Essen TA, Bos A, et al. Coagulopathy after hemorrhagic traumatic brain injury, an observational study of the incidence and prognosis. Acta Neurochir 2020;1(62):329–336; doi: 10.1007/s00701-019-04111-z PMC6982633 31741112

[4] Martini WZ, Chinkes DL, Pusateri AE, et al. Acute changes in fibrinogen metabolism and coagulation after hemorrhage in pigs. Am J Physiol Endocrinol Metab 2005;289(5):E930–E934; doi: 10.1152/ajpendo.00137.2005 15956050

[5] Martini WZ, Holcomb JB. Acidosis and coagulopathy: the differential effects on fibrinogen synthesis and breakdown in pigs. Ann Surg 2007;246(5):831–835; doi: 10.1097/SLA.0b013e3180cc2e94 17968176

[6] Lv K, Yuan Q, Fu P, et al. Impact of fibrinogen level on the prognosis of patients with traumatic brain injury: a single-center analysis of 2570 patients. World J Emerg Surg 2020;15(1):54; doi: 10.1186/s13017-020-00332-1 32977824 PMC7517804

[7] Spahn DR, Bouillon B, Cerny V, et al. The European guideline on management of major bleeding and coagulopathy following trauma: fifth edition. Crit Care 2019;23(1):98; doi: 10.1186/s13054-019-2347-3 30917843 PMC6436241

[8] Nakae R, Yokobori S, Takayama Y, et al. A retrospective study of the effect of fibrinogen levels during fresh frozen plasma transfusion in patients with traumatic brain injury. Acta Neurochir 2019;161(9):1943–1953; doi: 10.1007/s00701-019-04010-3 31309303

[9] Aubron C, Reade MC, Fraser JF, et al. Efficacy and safety of fibrinogen concentrate in trauma patients-a systematic review. J Crit Care 2014;29(3):471.e11–471.e17; doi: 10.1016/j.jcrc.2013.12.011 24508201

[10] Huijben JA, van der Jagt M, Cnossen MC, et al. Variation in blood transfusion and coagulation management in traumatic brain injury at the intensive care unit: a survey in 66 neurotrauma centers participating in the collaborative European neurotrauma effectiveness research in traumatic brain injury study. J Neurotrauma 2018;15(35):323–332; doi: 10.1089/neu.2017.5194 28825511

[11] Geeraerts T, Velly L, Abdennour L, et al. French Society of Anaesthesia; Intensive Care Medicine; in partnership with Association de neuro-anesthésie-réanimation de langue française (Anarlf); French Society of Emergency Medicine (Société Française de Médecine d'urgence (SFMU); Société française de neurochirurgie (SFN); Groupe francophone de réanimation et d'urgences pédiatriques (GFRUP); Association des Anesthésistes-Réanimateurs pédiatriques d'expression française (Adarpef). Management of severe traumatic brain injury (first 24 hours). Anaesth Crit Care Pain Med 2018;37(2):171–186; doi: 10.1016/j.accpm.2017.12.001 29288841

[12] Romero JM, Kelly HR, Delgado Almandoz JE, et al. Contrast extravasation on CT angiography predicts hematoma expansion and mortality in acute traumatic subdural hemorrhage. AJNR Am J Neuroradiol 2013;34(8):1528–1534; doi: 10.3174/ajnr.A3434 23449655 PMC8051476

[13] Letourneau-Guillon L, Huynh T, Jakobovic R, et al. Traumatic intracranial hematomas: prognostic value of contrast extravasation. AJNR Am J Neuroradiol 2013;34(4):773–779; doi: 10.3174/ajnr.A3309 23079406 PMC7964508

[14] Rosa M Jr, da Rocha AJ, Maia ACM Jr, et al. Contusion contrast extravasation depicted on multidetector computed tomography angiography predicts growth and mortality in traumatic brain contusion. J Neurotrauma 2016;33(11):1015–1022; doi: 10.1089/neu.2015.4062 26214242

[15] Orito K, Hirohata M, Nakamura Y, et al. Predictive value of leakage signs for pure brain contusional hematoma expansion. J Neurotrauma 2018;35(5):760–766; doi: 10.1089/neu.2017.5247 28967295

[16] Huang APH, Lee CW, Hsieh HJ, et al. Early parenchymal contrast extravasation predicts subsequent hemorrhage progression, clinical deterioration, and need for surgery in patients with traumatic cerebral contusion. J Trauma 2011;71(6):1593–1599; doi: 10.1097/TA.0b013e31822c8865 22182869

[17] Tartaglione M, Carenzo L, Gamberini L, et al. Multicentre observational study on practice of prehospital management of hypotensive trauma patients: the SPITFIRE study protocol. BMJ Open 2022;30;12(5):e062097; doi: 10.1136/bmjopen-2022-062097 PMC9152935 35636792

[18] Sperry JL, Guyette FX, Brown JB, et al. Prehospital plasma during air medical transport in trauma patients at risk for hemorrhagic shock. N Engl J Med 2018;26;379(4):315–326; doi: 10.1056/NEJMoa1802345 30044935

[19] Guidelines Committee on the Management of Severe Head Injury, Japan Society of Neurotraumatology. Guidelines for the Management of Severe Head Injury. 3rd ed. Igaku-Shoin Ltd: Tokyo, Japan; 2013.

[20] Shigemori M, Abe T, Aruga T, et al. Guidelines Committee on the Management of Severe Head Injury, Japan Society of Neurotraumatology. Guidelines for the Management of Severe Head Injury. 2nd ed. Guidelines from the Guidelines Committee on the Management of Severe Head Injury, the Japan Society of Neurotraumatology. Neurol Med Chir (Tokyo) 2012;52:1–30; doi: 10.2176/nmc.52.1 22278024

[21] Guidelines Committee on the Management of Severe Head Injury, Japan Society of Neurotraumatology. Guidelines for the Management of Severe Head Injury, 4th Ed. Igaku-Shoin Ltd: Tokyo, Japan; 2019.

[22] Marshall LE. A new classification of head injury based on computed tomography. J Neurol Surg 1991;75:S14–S20; doi: 10.3171/sup.1991.75.1s.0s14

[23] Jennett B, Bond M. Assessment of outcome after severe brain damage. Lancet 1975;1(7905):480–484; doi: 10.1016/S0140-6736(75)92830-5 46957

[24] Wafaisade A, Lefering R, Tjardes T, et al. Acute coagulopathy in isolated blunt traumatic brain injury. Neurocrit Care 2010;12(2):211–219; doi: 10.1007/s12028-009-9281-1 19806475

[25] McQuilten ZK, Wood EM, Bailey M, et al. Fibrinogen is an independent predictor of mortality in major trauma patients: a five-year statewide cohort study. Injury 2017;48(5):1074–1081; doi: 10.1016/j.injury.2016.11.021 28190583

[26] Ohmori T, Kitamura T, Tanaka K, et al. Admission fibrinogen levels in severe trauma patients: a comparison of elderly and younger patients. Injury 2015;46(9):1779–1783; doi: 10.1016/j.injury.2015.04.007 25943293

[27] Savioli G, Ceresa IF, Caneva L, et al. Trauma-induced coagulopathy: overview of an emerging medical problem from pathophysiology to outcomes. Medicines (Basel) 2021;24;8(4):16; doi: 10.3390/medicines8040016 PMC8064317 33805197

[28] Gee AC, Sawai RS, Differding J, et al. The influence of sex hormones on coagulation and inflammation in the trauma patient. Shock 2008;29(3):334–341; doi: 10.1097/shk.0b013e3181506ee5 18437714

[29] Kimura Y, Kimura S, Sumita S, et al. Predictors of hypofibrinogenemia in blunt trauma patients on admission. J Anesth 2015;29(2):242–248; doi: 10.1007/s00540-014-1895-6 25112812

[30] Hawryluk GWJ, Manley GT. Classification of traumatic brain injury: past, present, and future. Handb Clin Neurol 2015;127:15–21; doi: 10.1016/B978-0-444-52892-6.00002-7 25702207

[31] Zhang J, Zhang F, Dong JF. Coagulopathy induced by traumatic brain injury: systemic manifestation of a localized injury. Blood 2018;131(18):2001–2006; doi: 10.1182/blood-2017-11-784108 29507078 PMC5934798

[32] Nakae R, Takayama Y, Kuwamoto K, et al. Time course of coagulation and fibrinolytic parameters in patients with traumatic brain injury. J Neurotrauma 2016;33(7):688–695; doi: 10.1089/neu.2015.4039 26414158

[33] Toulon P, Ozier Y, Ankri A, et al. Point-of-care versus central laboratory coagulation testing during haemorrhagic surgery. a multicenter study. Thromb Haemost 2009;101(2):394–401; doi: 10.1160/TH08-06-0383 19190827

[34] Chang R, Folkerson LE, Sloan D, et al. Early plasma transfusion is associated with improved survival after isolated traumatic brain injury in patients with multifocal intracranial hemorrhage. Surgery 2017;161(2):538–545; doi: 10.1016/j.surg.2016.08.023 27776795 PMC5243198

[35] Watson GA, Sperry JL, Rosengart MR, et al. Fresh frozen plasma is independently associated with a higher risk of multiple organ failure and acute respiratory distress syndrome. J Trauma 2009;67(2):221–227; discussion, 228; doi: 10.1097/TA.0b013e3181ad5957 19667872

[36] Long B, April MD, Summers S, et al. Whole-body CT versus selective radiological imaging strategy in trauma: an evidence-based clinical review. Am J Emerg Med 2017;35(9):1356–1362; doi: 10.1016/j.ajem.2017.03.048 28366287

