# Supplementary material for: Association of Extravascular Leakage on Computed Tomography Angiography with Fibrinogen Levels at Admission in Patients with Traumatic Brain Injury
Source: Neurotrauma Rep. 2022 Dec 26;4(1):3–13. doi: 10.1089/neur.2022.0054 (PMC9811953; doi:10.1089/neur.2022.0054)
Supplement: Supplemental data [file Supp_TableS1.docx]

Table S1 Marshall classification of traumatic brain injury

| I. Diffuse injury | No visible intracranial pathology |
| --- | --- |
| II. Diffuse injury | Midline shift of 0 to 5 mm  Basal cisterns remain visible  No high or mixed density lesions > 25 cm^3^ |
| III. Diffuse injury | Midline shift of 0 to 5 mm  Basal cisterns compressed or completely effaced  No high or mixed density lesions > 25 cm^3^ |
| IV. Diffuse injury | Midline shift > 5 mm  No high or mixed density lesions > 25 cm^3^ |
| V. Evacuated mass lesion | Any lesion evacuated surgically |
| VI. Non evacuated mass lesion | High or mixed density lesions > 25 cm^3^  Not surgically evacuated |
