# Supplementary material for: Association of Extravascular Leakage on Computed Tomography Angiography with Fibrinogen Levels at Admission in Patients with Traumatic Brain Injury
Source: Neurotrauma Rep. 2022 Dec 26;4(1):3–13. doi: 10.1089/neur.2022.0054 (PMC9811953; doi:10.1089/neur.2022.0054)
Supplement: Supplemental data [file Supp_TableS2.docx]

Table S2. Glasgow Outcome Scale, definition of terms

| 1. | Dead |
| --- | --- |
| 2. | Vegetative state |
| 3. | Severe disability |
| 4. | Moderate disability |
| 5. | Good recovery |
