# Supplementary material for: Association of Extravascular Leakage on Computed Tomography Angiography with Fibrinogen Levels at Admission in Patients with Traumatic Brain Injury
Source: Neurotrauma Rep. 2022 Dec 26;4(1):3–13. doi: 10.1089/neur.2022.0054 (PMC9811953; doi:10.1089/neur.2022.0054)

Figure S1: Scatter plots of residuals

The scatter plots of the fibrinogen residuals showed normal distribution.


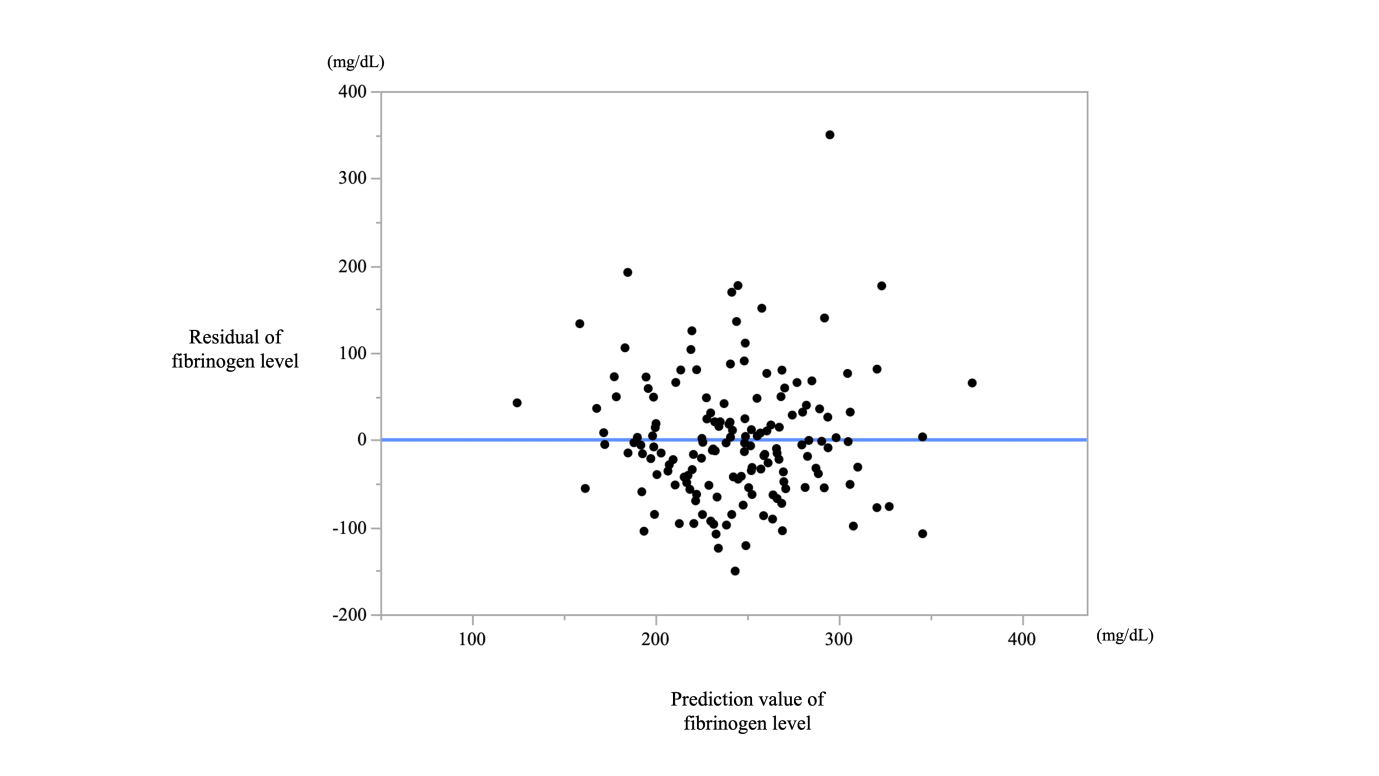

Supplement: Supplemental data [file Supp_FigS1.docx]
