# Supplementary material for: Association of Extravascular Leakage on Computed Tomography Angiography with Fibrinogen Levels at Admission in Patients with Traumatic Brain Injury
Source: Neurotrauma Rep. 2022 Dec 26;4(1):3–13. doi: 10.1089/neur.2022.0054 (PMC9811953; doi:10.1089/neur.2022.0054)
Supplement: Supplemental data [file Supp_TableS3.docx]

Table S3. Differences in blood test results and transfusion units between contrast extravasation and no contrast extravasation cases according to the emergent surgery subgroup

| Outcome | Contrast extravasation  (n = 34) | No contrast extravasation  (n = 39) | P value |
| --- | --- | --- | --- |
| Fibrinogen*, median (IQR); mg/dL | 180 (161–238) | 252 (185–345) | 0.004 |
| Minimum fibrinogen level within 24 h of admission, median (IQR); mg/dL | 116 (79–152) | 221 (139–273) | <0.0001 |
| Platelet count*, median (IQR) - ×10^3^/μL | 205 (152–229) | 214 (164–254) | 0.42 |
| PT-INR*, median (IQR) | 1.13 (1.04–1.21) | 1.11 (1.03–1.26) | 0.92 |
| aPTT*, median (IQR), s | 32.5 (26.5–38.3) | 28 (25–35) | 0.12 |
| Number of patients who received transfusions within 24 h of admission, n (%) |  |  |  |
| Red blood cell | 25 (73.5) | 18 (46.2) | 0.018 |
| Fresh frozen plasma | 28 (82.4) | 17 (43.6) | 0.0007 |
| Platelet concentrate | 13 (38.2) | 2 (5.13) | 0.0005 |
| Number of units transfused within 24 h of admission, median (IQR); units |  |  |  |
| Red blood cell | 8 (0–18) | 0 (0–4) | 0.0006 |
| Fresh frozen plasma | 14 (6–28) | 0 (0–6) | <0.0001 |
| Platelet concentrate | 0 (0–20) | 0 (0–0) | 0.0004 |

*Indicates values at admission

IQR, interquartile ratio; PT-INR, prothrombin time-international normalized ratio; aPTT: activated partial thromboplastin time
