# Supplementary material for: Association of Extravascular Leakage on Computed Tomography Angiography with Fibrinogen Levels at Admission in Patients with Traumatic Brain Injury
Source: Neurotrauma Rep. 2022 Dec 26;4(1):3–13. doi: 10.1089/neur.2022.0054 (PMC9811953; doi:10.1089/neur.2022.0054)
Supplement: Supplemental data [file Supp_DataS1.docx]

Supplementary Information

Scan parameters

SECT was performed using an X-ray tube voltage of 120 kV, and DECT was performed using X-ray tube voltages of 100 and 140 kV. The scan parameters for plain CT examination were quality reference mAs, 520 mAs; rotation time, 1 second; pitch, 0.25 mm/rotation; and collimation, 0.6 mm (128 slices). The scan parameters for contrast CT images obtained with SECT were as follows: quality reference mAs, 250 mAs; rotation time, 0.28 seconds; pitch, 0.55 mm/rotation; and collimation, 0.6 mm (128 slices). Those for contrast CT images obtained with DECT were quality reference mAs, 180 mAs; rotation time, 0.28 second; pitch, 0.9 mm/rotation; and collimation, 0.6 mm, 64 slices. Automatic tube current modulation (CARE Dose4D; Siemens) was used in all the cases.
